# Transcriptional coactivator NT‐PGC‐1*α* promotes gluconeogenic gene expression and enhances hepatic gluconeogenesis

**DOI:** 10.14814/phy2.13013

**Published:** 2016-10-26

**Authors:** Ji Suk Chang, Hee‐Jin Jun, Minsung Park

**Affiliations:** ^1^ Laboratory of Gene Regulation and MetabolismPennington Biomedical Research CenterBaton RougeLouisiana

**Keywords:** Diabetes, hepatic gluconeogenesis, NT‐PGC‐1alpha, obesity, PGC‐1alpha, Ppargc1a protein

## Abstract

The transcriptional coactivator PGC‐1*α* plays a central role in hepatic gluconeogenesis. We previously reported that alternative splicing of the PGC‐1*α* gene produces an additional transcript encoding the truncated protein NT‐PGC‐1*α*. NT‐PGC‐1*α* is co‐expressed with PGC‐1*α* and highly induced by fasting in the liver. NT‐PGC‐1*α* regulates tissue‐specific metabolism, but its role in the liver has not been investigated. Thus, the objective of this study was to determine the role of hepatic NT‐PGC‐1*α* in the regulation of gluconeogenesis. Adenovirus‐mediated expression of NT‐PGC‐1*α* in primary hepatocytes strongly stimulated the expression of key gluconeogenic enzyme genes (PEPCK and G6Pase), leading to increased glucose production. To further understand NT‐PGC‐1*α* function in hepatic gluconeogenesis in vivo, we took advantage of a previously reported FL‐PGC‐1*α* ^−/−^ mouse line that lacks full‐length PGC‐1*α* (FL‐PGC‐1*α*) but retains a slightly shorter and functionally equivalent form of NT‐PGC‐1*α* (NT‐PGC‐1*α* ^254^). In FL‐PGC‐1*α* ^−/−^ mice, NT‐PGC‐1*α* ^254^ was induced by fasting in the liver and recruited to the promoters of PEPCK and G6Pase genes. The enrichment of NT‐PGC‐1*α* ^254^ at the promoters was closely associated with fasting‐induced increase in PEPCK and G6Pase gene expression and efficient production of glucose from pyruvate during a pyruvate tolerance test in FL‐PGC‐1*α* ^−/−^ mice. Moreover, FL‐PGC‐1*α* ^−/−^ primary hepatocytes showed a significant increase in gluconeogenic gene expression and glucose production after treatment with dexamethasone and forskolin, suggesting that NT‐PGC‐1*α* ^254^ is sufficient to stimulate the gluconeogenic program in the absence of FL‐PGC‐1*α*. Collectively, our findings highlight the role of hepatic NT‐PGC‐1*α* in stimulating gluconeogenic gene expression and glucose production.

## Introduction

Blood glucose levels are maintained by a tight regulation of glucose uptake by peripheral tissues and glucose production in the liver. During prolonged fasting, liver induces the gluconeogenic pathway that synthesizes glucose from non‐carbohydrate precursors such as pyruvate, lactate, glycerol and alanine. This metabolic process is primarily controlled by key gluconeogenic enzymes such as PEPCK and G6Pase, the activities of which are regulated at the transcriptional level through hormonal and nutrient signals (Pilkis and Granner 1992).

The PGC‐1*α* transcriptional coactivator is a master regulator that links fasting‐induced hormonal and nutrient signals to the gluconeogenic gene transcription. Upon glucose deprivation, hepatic PGC‐1*α* expression is highly induced by fasting‐induced glucagon and glucocorticoids that activate the cAMP/PKA/CREB and glucocorticoid receptor pathways, respectively (Herzig et al. 2001; Yoon et al. 2001). Moreover, PGC‐1*α* protein levels are elevated by posttranslational modifications including phosphorylation and O‐GlcNAcylation that protect PGC‐1*α* from degradation (Puigserver et al. 2001; Cao et al. 2004; Ruan et al. 2012). A fasting‐induced increase in PGC‐1*α* expression and activity in turn results in coactivation of FOXO1, HNF4*α*, and glucocorticoid receptor (GR), which cooperatively promote the full activation of PEPCK and G6Pase gene expression (Imai et al. 1990; Herzig et al. 2001; Nakae et al. 2001; Yoon et al. 2001; Rhee et al. 2003).

**Figure 1:**
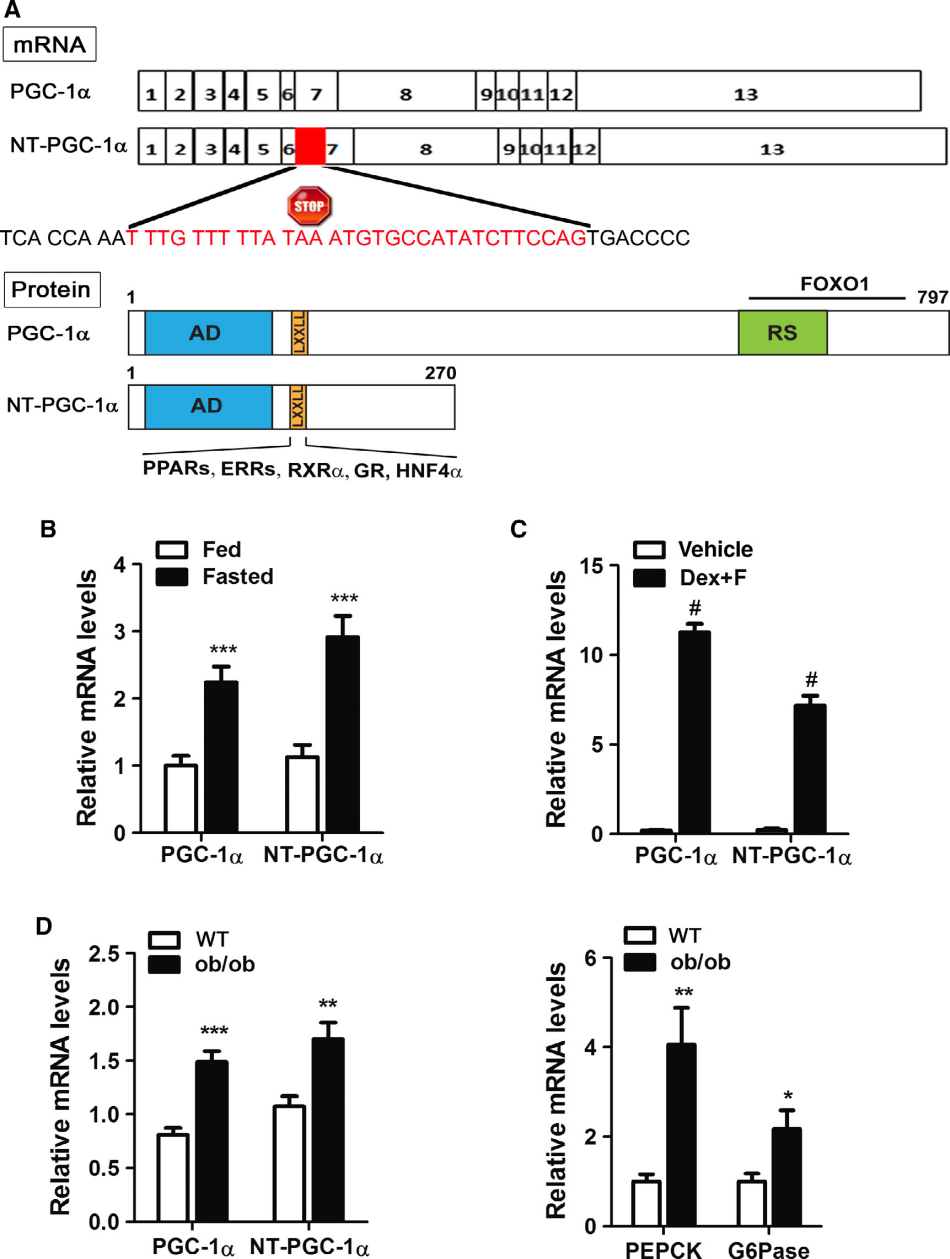
Hepatic NT‐PGC‐1*α* expression in normal and diabetic mice. (A) Schematic diagram of PGC‐1*α* and NT‐PGC‐1*α* transcripts and proteins. A gray box between exon 6 and exon 7 represents 31 bp intron sequences inserted by alternative 3′ splicing of the PGC‐1*α* gene. The alternatively spliced transcript produces a truncated protein termed NT‐PGC‐1*α* due to the presence of a premature stop codon. (B) Induction of hepatic PGC‐1*α* and NT‐PGC‐1*α* mRNA expression by fasting in C57BL/6J mice (*n* = 8). Eleven‐week‐old mice were fed ad libitum or fasted for 24 h before tissue collection. Expression of hepatic PGC‐1*α* and NT‐PGC‐1*α* mRNA was analyzed using isoform‐specific primers (Chang et al. 2012). (C) Regulation of PGC‐1*α* and NT‐PGC‐1*α* mRNA expression in primary hepatocytes. Mouse primary hepatocytes were established from C57BL/6J mice and treated with vehicle or 1 μmol/L dexamethasone (Dex) and 10 μmol/L forskolin (F) for 2 h. (D) Abnormal elevation of hepatic PGC‐1*α* and NT‐PGC‐1*α* gene expression in ob/ob mice. 17‐week‐old ob/ob male mice and their WT male littermates (*n* = 7) were fed ad libitum before tissue collection. Data represent mean ± SEM. *P < 0.05; **P < 0.01; ***P < 0.001; ^#^ *P* < 0.0001.

The PGC‐1*α* gene was originally thought to produce a single protein consisting of 797 amino acids, but recent studies have established that multiple mRNAs and proteins are generated by alternative splicing events in a tissue‐specific manner (Miura et al. 2008; Zhang et al. 2009; Chang et al. 2010; Tadaishi et al. 2011; Ruas et al. 2012). Alternative 3′ splicing between exons 6 and 7 of the PGC‐1*α* gene introduces an in‐frame stop codon into the PGC‐1*α* transcript, thus producing a shorter protein termed N‐terminal PGC‐1*α* (NT‐PGC‐1*α*, 270 aa) (Fig. 1A) (Zhang et al. 2009). NT‐PGC‐1*α* is co‐expressed with PGC‐1*α* in metabolically active tissues from mice to humans and its expression is highly induced by nutritional and environmental stimuli which activate the PGC‐1*α* gene (Zhang et al. 2009). Although it lacks the C‐terminal domain of PGC‐1*α*, NT‐PGC‐1*α* retains the transcriptional activation and nuclear receptor interaction domains necessary for coactivation of a number of nuclear receptors (Zhang et al. 2009; Chang et al. 2010, 2012). NT‐PGC‐1*α* regulates tissue‐specific metabolism; it regulates adaptive thermogenesis in brown adipose tissue (Zhang et al. 2009; Chang et al. 2010; Jun et al. 2012), angiogenesis in skeletal muscle (Thom et al. 2014), and is implicated in the pathogenesis of Huntington's disease (Johri et al. 2011; Soyal et al. 2012). In the liver, NT‐PGC‐1*α* expression is highly elevated by fasting (Zhang et al. 2009), but its hepatic role has not been elucidated. We here investigated the hypothesis that NT‐PGC‐1*α*‐mediated induction of gluconeogenic gene expression contributes to fasting‐induced hepatic glucose production upon glucose deprivation.

## Material and Methods

### Mice

All animal handling and experiments were conducted according to the procedures reviewed and approved by the Pennington Biomedical Research Center Institutional Animal Care and Use Committee (IACUC). FL‐PGC‐1*α* ^−/−^ mice deficient in full‐length PGC‐1*α* (FL‐PGC‐1*α*) have been described previously (Leone et al. 2005; Chang et al. 2012; Jun et al. 2014). The PGC‐1*α* gene product produced in FL‐PGC‐1*α* ^−/−^ mice was defined as NT‐PGC‐1*α* ^254^ to distinguish it from naturally occurring NT‐PGC‐1*α* (Chang et al. 2012; Jun et al. 2014). Ob/ob male mice (B6.Cg‐Lepob/J#000632) and +/? (WT) littermates were purchased from Jackson Laboratory. All mice were housed on a 12 h light/12 h dark cycle. Male mice were either fed ad libitum a normal chow diet or subjected to a 24‐h fast but with free access to water.

### Primary hepatocyte isolation and culture

Primary mouse hepatocytes were isolated as described previously with slight modifications (Klaunig et al. 1981; Zhang et al. 2012). Briefly, mice were anesthetized with isoflurane and the inferior vena cava was cannulated with a 23G × 1 inch needle. The portal vein was immediately cut to allow fluid to drain. Liver perfusion was initiated with Hank's buffered salt solution (HBSS) containing 0.5 mmol/L EGTA, followed by DMEM medium containing 5 mmol/L glucose, penicillin/streptomycin, 15 mmol/L HEPES, 100 U/mL collagenase (Type IV, Worthington, Lakewood, NJ). After sufficient digestion, the liver was excised and placed in the same digestion medium and torn and shaken gently with forceps to liberate the cells. The cell suspension was then filtered through a 70 μm cell strainer, washed three times by centrifugation at 50 × *g* for 5 min at 4°C, and then resuspended in DMEM/F‐12 medium supplemented with 25 mmol/L glucose, 10% FBS, 15 mmol/L HEPES, 100 nmol/L dexamethasone, and penicillin/streptomycin. Hepatocytes were plated on collagen‐coated 12‐well plates and allowed to attach for 1 h at 37°C in a humidified incubator. Cells were washed once with DMEM medium containing 5 mmol/L glucose and incubated in the same medium supplemented with 10% FBS, 100 nmol/L dexamethasone, 1 nmol/L insulin, and penicillin/streptomycin for 4 h. The medium was then replaced with serum‐free DMEM medium supplemented with 5 mmol/L glucose, 10 nmol/L dexamethasone, 1 nmol/L insulin, and penicillin/streptomycin. After an overnight incubation, cells were cultured in the same medium containing 10% FBS. When mimicking a fasted condition, cells were treated with or without 1 μmol/L dexamethasone and 10 μmol/L forskolin for 2 h in serum‐free DMEM medium supplemented with 5 mmol/L glucose and penicillin/streptomycin.

### Adenovirus production and infection

The cDNA encoding NT‐PGC‐1*α*‐HA or PGC‐1*α*‐HA was cloned into pAdEasy‐GFP shuttle vector (Clontech, Mountain View, CA). Recombination of AdEasy‐GFP, AdEasy‐NT‐PGC‐1*α*‐HA, or AdEasy‐PGC‐1*α*‐HA with adenoviral gene carrier vector was performed by transformation into BJ5183‐AD‐1 competent cells (Agilent Technologies, Santa Clara, CA). Recombinant adenovirus was produced by transfecting recombinant adenoviral plasmids into HEK293 cells (Luo et al. 2007), amplified in HEK293 cells, and purified using an adenovirus purification kit (Virapur, San Diego, CA). Titers of GFP‐tagged adenoviruses were determined by the flow cytometric method as described previously (Hitt et al. 2000). Briefly, the percentage of GFP‐expressing cells was assessed for each viral dilution in triplicate wells and the functional viral concentration (GFU) was defined by the following formula: GFU/mL = (percent GFP positive cells) × (number of cells per well)/(volume per well) × (viral dilution factor). Primary hepatocytes were infected with adenovirus in serum‐free DMEM medium supplemented with 5 mmol/L glucose, 100 nmol/L dexamethasone, and 1 nmol/L insulin. After a 16 h‐infection, cells were replaced with the same media containing 10% FBS and incubated for additional 7 h with or without addition of 10 nmol/L insulin prior to collection for gene expression analysis.

### Glucose production assay

Primary hepatocytes were cultured for 8 h in the presence and absence of 1 μmol/L dexamethasone and 10 μmol/L forskolin in glucose‐ and serum‐free DMEM medium supplemented with 2 mmol/L sodium pyruvate and 20 mmol/L sodium lactate. In case of adenovirus‐infected primary hepatocytes, cells were cultured for 3 h in the same medium. The glucose concentration in the medium was measured using a colorimetric glucose assay kit (Sigma, St. Louis, MO) and normalized to the total protein content determined from the whole cell extracts.

### Co‐immunoprecipitation assay

NT‐PGC‐1*α*‐HA and Flag‐HNF4*α* were co‐transfected into HEK293 cells using Fugene 6 (Roche, Indianapolis, IN). Immunoprecipitation assays were carried out as described previously by Chang et al. (2010). Briefly, whole cell extracts were incubated with Flag antibody (Sigma) or HA antibody (Abcam, Cambridge, MA) overnight at 4°C, followed by incubation with protein G‐agarose beads. The immunoprecipitates were extensively washed, separated by SDS‐PAGE, and immunoblotted with HA or Flag antibody.

### Luciferase reporter assay

HEK293 cells were transiently transfected with pHNF4‐tk‐luc (Yoshida et al. 1997), pcDNA‐Flag‐HNF4*α*, and pcDNA‐NT‐PGC‐1*α*‐HA. pRL‐SV40 control plasmid expressing *Renilla* luciferase was used for normalization. The firefly luciferase activity was determined 48 h after transfection using a Promega Dual‐Luciferase assay kit (Promega, Madison, WI) and normalized using *Renilla* luciferase activity. Data represent mean ± SEM of at least three independent experiments.

### Chromatin immunoprecipitation assays

The ChIP assays were performed as described previously (Chang et al. 2012). Briefly, after an overnight fast, mouse livers were chopped into small pieces, fixed with formaldehyde, homogenized, and then sonicated. After centrifugation, the nuclear extracts were immunoprecipitated with rabbit polyclonal antibody directed against the N‐terminus of PGC‐1*α* (Zhang et al. 2009) or anti‐rabbit IgG (Santa Cruz Biotechnology). After de‐crosslinking of the precipitated DNA, samples were analyzed by PCR amplification using the following primers: PEPCK (−538 to −284), 5′‐AATCCACCACACACCTAGTGAGG‐3′ and 5′‐TTGACCCTGCCTGTTGCTGATG‐3′; G6Pase (−401 to −195), 5′‐CAGTAGCAAACTTCCCTTTGTCTTC‐3′ and 5′‐CAAAAACAGCCTGATCGCCATT‐3′. A nontargeting intragenic region of PEPCK and G6Pase was amplified using the following primers: PEPCK (+1753 to +1884), 5′‐CTCGGATGGGCATATCTGTGCTG‐3′ and 5′‐ CAACTTCTCCCTCCACCTTCGAT‐3′; G6Pase (+2592 to +2780), 5′‐AAGTGCTGGGATTACAGGTGTGAG‐3′ and 5′‐AGTCTGAGGCTTGAGGATTATAAACTG‐3′.

### Pyruvate tolerance test

Following a 16 h fast, mice received an intraperitoneal injection of 1.75 g/kg sodium pyruvate dissolved in PBS. Blood glucose concentrations were measured via tail bleed using OneTouch blood glucose meter (LifeScan, Chesterbrook, PA) at different time points (0, 15, 30, 60, and 120 min).

### Quantitative real‐time PCR analysis

Total RNA from livers and hepatocytes was extracted and reverse‐transcribed as described previously (Chang et al. 2010, 2012). cDNA was analyzed by quantitative real‐time PCR analysis using a SYBR green PCR kit (Bio‐Rad, Hercules, CA) and an Applied Biosystems 7900 (Applied Biosystems, Carlsbad, CA). Relative mRNA expression of the genes of interest was determined after normalization to cyclophilin by the ΔΔCt method.

### Western blot analysis

Whole cell extracts were prepared from tissues and hepatocytes by homogenization in lysis buffer (Chang et al. 2010) and subjected to western blot analysis using following antibodies: anti‐PGC‐1*α* (Zhang et al. 2009) and anti‐*β*‐actin (Sigma). Protein concentration was determined using Bio‐Rad DC protein assay reagents according to the manufacturer's instructions.

### Statistical analysis

All data are presented as mean ± SEM. Student *t* test or one‐way ANOVA was used to compare the difference between groups using Graphpad Prism 5 software. Values of *P *<* *0.05 were considered statistically significant. ^*^ *P *<* *0.05, ^**^ *P *<* *0.01, ^***^ *P *<* *0.001, ^*#*^ *P *<* *0.0001.

## Results

### Hepatic NT‐PGC‐1*α* expression is elevated by fasting in normal lean mice and abnormally induced in diabetic mice

PGC‐1*α* is upregulated by fasting in the liver and promotes the expression of gluconeogenic genes by co‐activating FOXO1, HNF4*α*, and glucocorticoid receptor (GR) (Imai et al. 1990; Herzig et al. 2001; Nakae et al. 2001; Yoon et al. 2001; Rhee et al. 2003). We previously reported that NT‐PGC‐1*α* is co‐expressed with PGC‐1*α* in the liver and its mRNA and protein expression is highly elevated by fasting (Zhang et al. 2009). Figure 1B shows that PGC‐1*α* and NT‐PGC‐1*α* mRNA levels are significantly higher in the fasted livers than in the fed livers. To test whether elevated expression of NT‐PGC‐1*α* is dependent on fasting‐induced glucagon and glucocorticoid signaling pathways, primary hepatocytes were isolated from C57BL/6J mice and treated with forskolin and dexamethasone that mimic the action of glucagon and glucocorticoid, respectively. Following treatments with forskolin and dexamethasone, NT‐PGC‐1*α* expression was robustly induced in primary hepatocytes and its induction was comparable to that of PGC‐1*α* (Fig. 1C), indicating that expression of both isoforms are regulated by the same signaling pathways.

Abnormal upregulation of hepatic PGC‐1*α* gene has been implicated in the pathogenesis of hyperglycemia in mouse models of obesity and diabetes (ob/ob, db/db, and high‐fat‐fed mice) (Herzig et al. 2001; Yoon et al. 2001; Biddinger et al. 2005; Tamura et al. 2007; Estall et al. 2009; Rodgers et al. 2010). Thus, we tested whether hepatic NT‐PGC‐1*α* is upregulated in ob/ob mice. Indeed, NT‐PGC‐1*α* expression was 1.7‐fold‐increased in non‐fasting ob/ob mice compared to lean littermates (Fig. 1D, left panel). In addition, this induction was comparable to that of PGC‐1*α* (1.8‐fold increase). PEPCK and G6Pase were upregulated in ob/ob mice concomitantly with elevated expression of PGC‐1*α* and NT‐PGC‐1*α* (Fig. 1D, right panel).

### NT‐PGC‐1*α* interacts with HNF4*α* and enhances HNF4*α*‐mediated gene transcription

**Figure 2:**
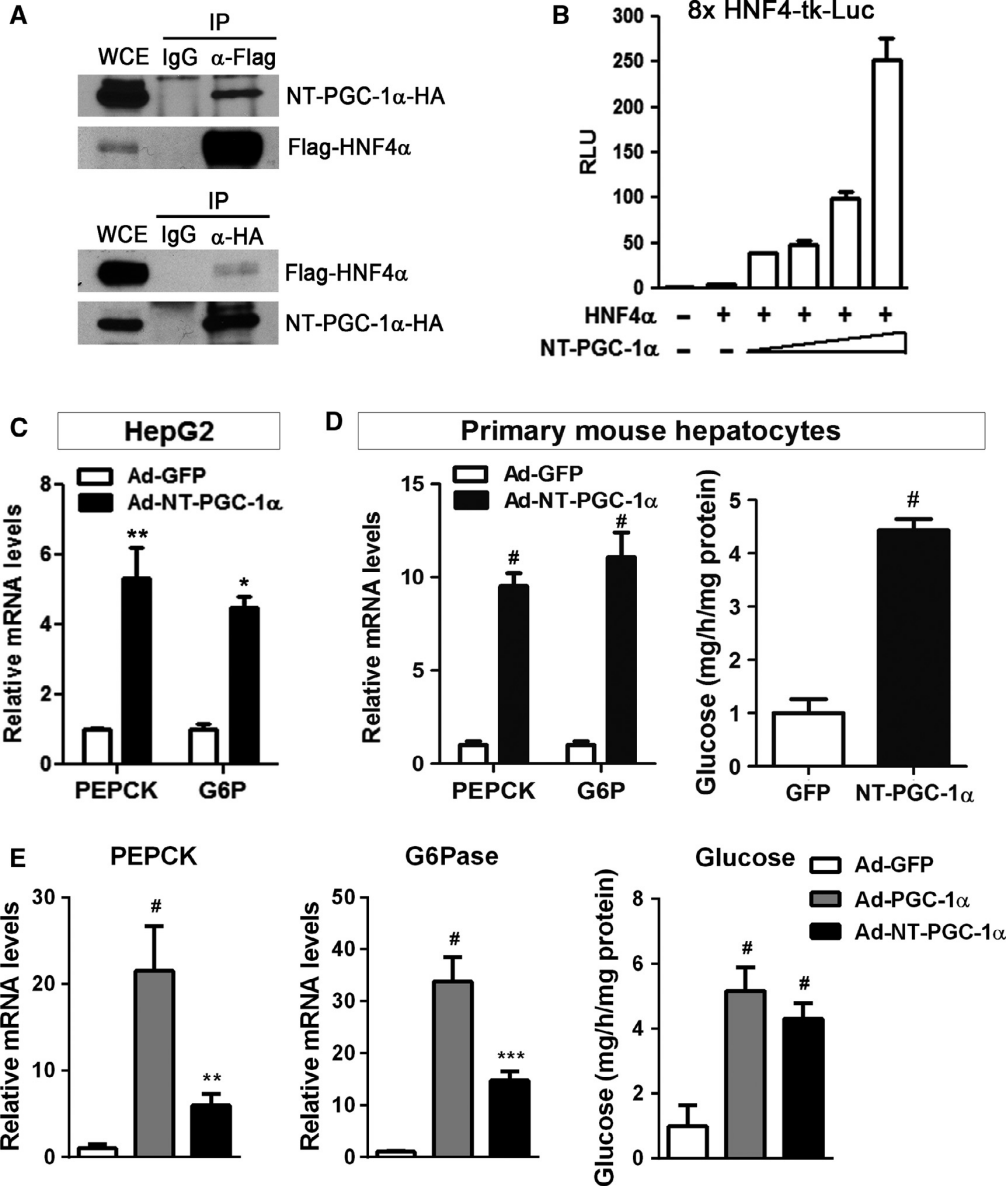
NT‐PGC‐1*α* stimulates gluconeogenic gene expression and glucose production in primary hepatocytes. (A) Interaction of NT‐PGC‐1*α* with HNF4*α*. Reciprocal co‐immunoprecipitation was carried out from HEK293 cells expressing NT‐PGC‐1*α*‐HA and Flag‐HNF4*α*. WCE, whole cell extracts. Reassembly of noncontinuous bands is demarcated by white spaces. (B) NT‐PGC‐1*α* activates HNF4*α*‐mediated transcription. HEK293 cells were transfected with a luciferase reporter gene containing eight copies of HNF4*α* binding sites, HNF4*α*, and increasing amounts of NT‐PGC‐1*α*. Luciferase activity was determined 48 h after transfection and normalized using *Renilla* luciferase activity. Data represent mean ± SEM of at least three independent experiments. (C) NT‐PGC‐1*α* induces expression of PEPCK and G6Pase genes. HepG2 cells were transduced with recombinant adenovirus carrying the cDNA encoding mouse NT‐PGC‐1*α* (Ad‐NT‐PGC‐1*α*) or control adenovirus expressing GFP (Ad‐GFP) for 16 h. Relative mRNA levels of PEPCK and G6Pase were determined by qPCR. (D) NT‐PGC‐1*α* stimulates gluconeogenic gene expression and glucose production. Mouse primary hepatocytes were transduced with Ad‐NT‐PGC‐1*α* or Ad‐GFP adenovirus for 16 h and analyzed for gluconeogenic gene expression and glucose production. (E) Effects of PGC‐1*α* and NT‐PGC‐1*α* on gluconeogenic gene expression and glucose production. Mouse primary hepatocytes were transduced with Ad‐PGC‐1*α*, Ad‐NT‐PGC‐1*α* or Ad‐GFP adenovirus for 16 h and analyzed for gluconeogenic gene expression and glucose production. Data represent mean ± SEM. *P < 0.05; **P < 0.01; ^#^ *P* < 0.0001.

Hepatic PGC‐1*α* co‐activates FOXO1, HNF4*α*, and GR bound in the multiple sites of the PEPCK and G6Pase promoters, thus mediating its synergistic effect on transcriptional activation of PEPCK and G6Pase promoters (Imai et al. 1990; Herzig et al. 2001; Nakae et al. 2001; Yoon et al. 2001; Rhee et al. 2003). The truncated NT‐PGC‐1*α* protein retains the sequences (LXXLL) necessary for binding to nuclear receptors such as HNF4*α* and GR but not the C‐terminal domain required for binding to FOXO1 (Fig. 1A). Thus, we hypothesized that NT‐PGC‐1*α* may co‐activate HNF4*α* and GR in the liver. Especially, HNF4*α* is a liver‐enriched orphan nuclear receptor that plays a crucial role in the regulation of PEPCK and G6Pase genes as well as a large number of hepatocyte‐specific genes (Boustead et al. 2003; Rhee et al. 2003). HNF4*α* knockout abolishes PGC‐1*α*‐dependent induction of PEPCK and G6Pase gene expression in vivo (Rhee et al. 2003). To test our hypothesis, we analyzed the interaction between NT‐PGC‐1*α* and HNF4*α* using co‐immunoprecipitation assay by transfecting HEK293 cells with NT‐PGC‐1*α*‐HA and Flag‐HNF4*α*. Immunoprecipitation of Flag‐HNF4*α* with Flag antibody co‐precipitated NT‐PGC‐1*α*‐HA (Fig. 2A, top panel). Reciprocally, Flag‐HNF4*α* was co‐immunoprecipitated with NT‐PGC‐1*α*‐HA by immunoprecipitation with HA antibody (Fig. 2A, bottom panel). Next, to determine whether NT‐PGC‐1*α* increases transcriptional activity of HNF4*α*, we performed transient transfection and luciferase reporter assays using a HNF4*α*‐responsive luciferase reporter construct containing multiple HNF4*α* binding sites (8x HNF4‐tk‐Luc) (Yoshida et al. 1997). HNF4*α* alone did not result in reporter gene expression, whereas co‐transfection of HNF4*α* with NT‐PGC‐1*α* robustly increased HNF4*α*‐mediated transcription of the luciferase reporter in a dose‐dependent manner (Fig. 2B).

### NT‐PGC‐1*α* induces gluconeogenesis in primary hepatocytes

To assess whether NT‐PGC‐1*α* activates transcription of endogenous gluconeogenic genes, we transduced HepG2 hepatoma cells with recombinant adenovirus carrying the cDNA encoding mouse NT‐PGC‐1*α* (Ad‐NT‐PGC‐1*α*) or control adenovirus expressing GFP (Ad‐GFP). HNF4*α* is abundantly expressed in HepG2 cells, but its transcriptional activity is very low due to low expression of the PGC‐1*α* gene (Martinez‐Jimenez et al. 2006). Adenovirus‐mediated expression of NT‐PGC‐1*α* in HepG2 cells strongly stimulated the expression of PEPCK and G6Pase genes compared to Ad‐GFP control (Fig. 2C). Similarly, NT‐PGC‐1*α* led to robust induction of PEPCK and G6Pase gene expression in mouse primary hepatocytes (Fig. 2D). A significant increase in PEPCK and G6Pase gene expression was accompanied by increased secretion of glucose from primary hepatocytes (Fig. 2D), clearly demonstrating that NT‐PGC‐1*α* activates gluconeogenesis in hepatocytes. Next, the effect of NT‐PGC‐1*α* on gluconeogenesis was compared with that of PGC‐1*α* by infecting primary hepatocytes with adenovirus expressing PGC‐1*α* and NT‐PGC‐1*α*. PGC‐1*α* led to higher induction of PEPCK and G6Pase gene expression than NT‐PGC‐1*α* (Fig. 2E, left and middle panels). However, glucose production driven by PGC‐1*α* and NT‐PGC‐1*α* was comparable; glucose production was not correlated with PEPCK and G6Pase mRNA levels in PGC‐1*α*‐ and NT‐PGC‐1*α*‐expressing hepatocytes (Fig. 2E, right panel). This could be because hepatocytes have a limited capacity to generate energy that is required for supporting gluconeogenesis so excessive PEPCK and G6Pase mRNA no longer enhance gluconeogenesis. To evaluate the importance of NT‐PGC‐1*α* in gluconeogenesis, we infected hepatocytes with retrovirus expressing an NT‐PGC‐1*α* shRNA (5′‐ATAAATGTGCCATATCTTCCA‐3′) that targets the 31 bp insert arising from alternative splicing of the PGC‐1*α* gene (Fig. 1A). The 31 bp insert is the only sequence unique to NT‐PGC‐1*α* mRNA. However, this shRNA sequence was not effective in suppressing NT‐PGC‐1*α* expression (data not shown). Commercial algorithms also predicted that this sequence is not effectively targeted to NT‐PGC‐1*α* mRNA.

### NT‐PGC‐1*α* activity is not directly regulated by insulin

**Figure 3:**
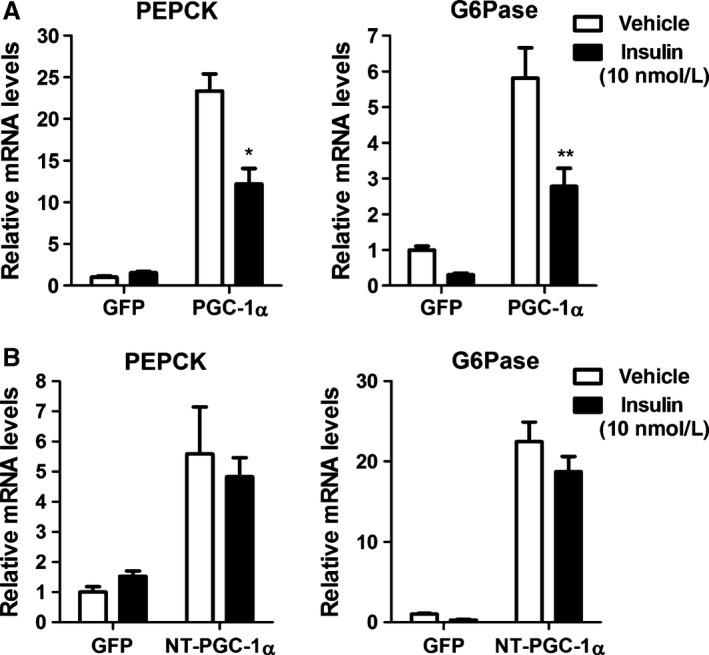
NT‐PGC‐1*α* activity is not inhibited by insulin. (A, B) Effect of insulin on PGC‐1*α*‐ and NT‐PGC‐1*α*‐mediated stimulation of PEPCK and G6Pase gene expression. Mouse primary hepatocytes were transduced with Ad‐PGC‐1*α*, Ad‐NT‐PGC‐1*α*, or Ad‐GFP adenovirus for 16 h, followed by incubation with or without 10 nmol/L insulin for 7 h. Relative mRNA levels of PEPCK and G6Pase were determined by qPCR. Data represent mean ± SEM. *P < 0.05; **P < 0.01.

Insulin inhibits PGC‐1*α* activity by Akt‐ and Cdc2‐like kinase 2 (CLK2)‐mediated phosphorylation (Li et al. 2007; Rodgers et al. 2010). These phosphorylations at the C‐terminal arginine/serine (RS)‐rich domain of PGC‐1*α* disrupt the interaction with FOXO1, resulting in repression of gluconeogenic gene expression and hepatic glucose production. Given that NT‐PGC‐1*α* lacks the C‐terminal domain of PGC‐1*α* (Fig. 1A), we investigated the differential response of PGC‐1*α* and NT‐PGC‐1*α* to insulin in primary hepatocytes. PGC‐1*α*‐mediated induction of PEPCK and G6Pase gene expression was significantly reduced by approximately 48% and 52%, respectively, after treatment with insulin (Fig. 3A). In contrast, insulin had no effect on NT‐PGC‐1*α*‐mediated induction of PEPCK and G6Pase gene expression (Fig. 3B). The results indicate that NT‐PGC‐1*α* activity is not directly regulated by insulin‐induced signaling and that NT‐PGC‐1*α*‐mediated gluconeogenic gene expression is not dependent on FOXO1.

### Hepatic NT‐PGC‐1*α* ^254^ is recruited to the PEPCK and G6Pase promoters in response to fasting in FL‐PGC‐1*α* ^−/−^ mice

**Figure 4:**
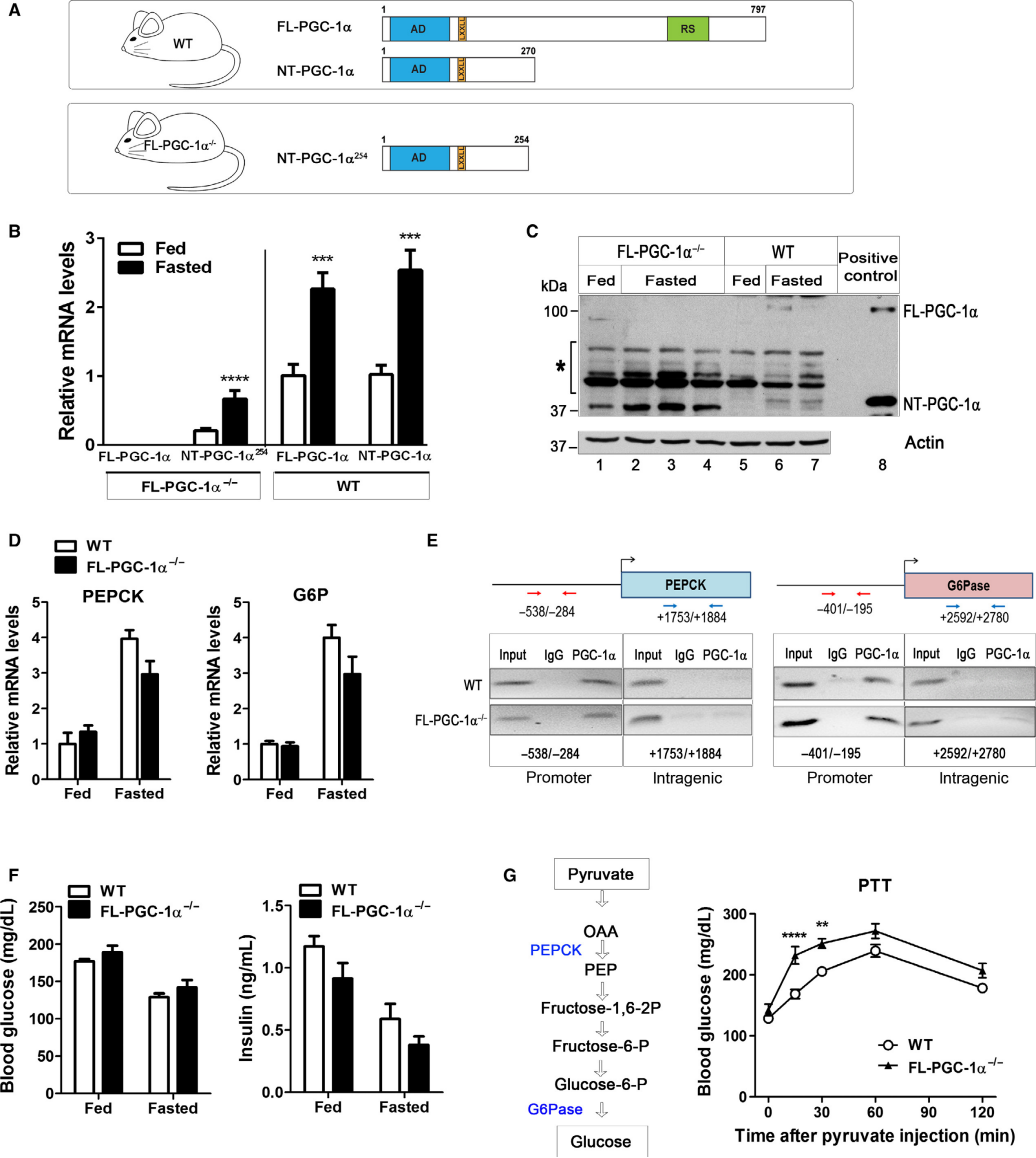
Fasting‐inducible NT‐PGC‐1*α* ^254^ is sufficient to activate the gluconeogenic program in FL‐PGC‐1*α* ^−/−^ mice. (A) Mouse lines used in the present studies. (B) Quantitative real‐time PCR and (C) Western blot analyses of hepatic PGC‐1*α* and NT‐PGC‐1*α* in WT mice and NT‐PGC‐1*α* ^254^ in FL‐PGC‐1*α* ^−/−^ mice. Eleven‐week old mice were fed ad libitum or fasted for 24 h before tissue collection. *, nonspecific bands. Reassembly of noncontinuous bands is demarcated by white spaces. (D) Quantitative real‐time PCR analysis of gluconeogenic gene expression in the fed and fasted livers. (E) Enrichment of NT‐PGC‐1*α* ^254^ protein in the PEPCK and G6Pase promoters. ChIP assays were carried out using a PGC‐1*α* antibody (Zhang et al. 2009; Jun et al. 2014) from the livers of WT and FL‐PGC‐1*α* ^−/−^ mice fasted for overnight. Representative images were shown from at least three independent ChIP assays. Reassembly of noncontinuous bands is demarcated by white spaces and black lines. (F) Normal glucose homeostasis in FL‐PGC‐1*α* ^−/−^ mice. Blood glucose and insulin levels of 11‐week‐old WT and FL‐PGC‐1*α* ^−/−^ mice fed or fasted for 24 h (*n* = 8–10). (G) Pyruvate tolerance test. Ten‐week‐old WT and FL‐PGC‐1*α* ^−/−^ mice (*n* = 8) were fasted for 16 h before receiving i.p. injection of a pyruvate solution. Data represent mean ± SEM. *P < 0.05; **P < 0.01; ***P < 0.001; ****P < 0.0001.

FL‐PGC‐1*α* ^−/−^ mice have been used as a unique mouse model to understand NT‐PGC‐1*α* function in vivo in the absence of full‐length PGC‐1*α* (FL‐PGC‐1*α*) (Chang et al. 2012; Jun et al. 2014; Kim et al. 2016). FL‐PGC‐1*α* ^−/−^ mice express a slightly shorter but functionally equivalent form of NT‐PGC‐1*α* (NT‐PGC‐1*α* ^254^) (Fig. 4A). NT‐PGC‐1*α* ^254^ retains the transcription activation and nuclear receptor interaction domains of NT‐PGC‐1*α* and is able to co‐activate a number of nuclear receptors as efficiently as NT‐PGC‐1*α* (Chang et al. 2012; Jun et al. 2014; Kim et al. 2016).

The FL‐PGC‐1*α* ^−/−^ liver expressed NT‐PGC‐1*α* ^254^ while lacking FL‐PGC‐1*α* (Fig. 4B). A 24 h fast led to a significant increase in NT‐PGC‐1*α* ^254^ mRNA expression in FL‐PGC‐1*α* ^−/−^ mice. NT‐PGC‐1*α* ^254^ protein expression was also elevated by fasting in FL‐PGC‐1*α* ^−/−^ mice (Fig. 4C, lanes 1–4). Similarly, FL‐PGC‐1*α* and NT‐PGC‐1*α* were significantly induced by fasting in WT mice (Fig. 4B and C, lanes 5–7). Despite less transcript levels of NT‐PGC‐1*α* ^254^ compared to NT‐PGC‐1*α*, protein levels of NT‐PGC‐1*α* ^254^ were significantly higher than those of NT‐PGC‐1*α* in the fed and fasted conditions. We reasoned that the increase in NT‐PGC‐1*α* ^254^ protein expression might occur as a mechanism to compensate for the loss of FL‐PGC‐1*α* as previously seen in brown adipose tissue (Chang et al. 2012). Of note is that expression of two other PGC‐1*α* family members, PGC‐1*β* and PRC, was not altered in FL‐PGC‐1*α* ^−/−^ mice as previously reported by Leone et al. (2005) (data not shown).

Elevated expression of NT‐PGC‐1*α* ^254^ was closely associated with a fasting‐induced increase in PEPCK and G6Pase gene expression in FL‐PGC‐1*α* ^−/−^ mice (Fig. 4D). To determine whether fasting‐induced NT‐PGC‐1*α* ^254^ protein regulates PEPCK and G6Pase gene expression by being recruited to the promoters of PEPCK and G6Pase genes in FL‐PGC‐1*α* ^−/−^ mice, we employed a chromatin immunoprecipitation (ChIP) assay using a PGC‐1*α* antibody. The PGC‐1*α* antibody has been confirmed for its specificity to immunoprecipitate FL‐PGC‐1*α* and NT‐PGC‐1*α* (WT mice) and NT‐PGC‐1*α* ^254^ (FL‐PGC‐1*α* ^−/−^ mice) (Jun et al. 2014). In the fasted WT livers, FL‐PGC‐1*α* and NT‐PGC‐1*α* were enriched at the PEPCK and G6Pase gene promoters containing binding sites for HNF4*α* and GR (Imai et al. 1990; Hall et al. 1995; Yoon et al. 2001; Rhee et al. 2003; Vander Kooi et al. 2005) (Fig. 4E). No binding of FL‐PGC‐1*α* and NT‐PGC‐1*α* was detected at nontargeting intragenic regions of PEPCK and G6Pase genes, strengthening their specific recruitment to the promoters for activation of PEPCK and G6Pase gene expression. Similarly, NT‐PGC‐1*α* ^254^ was enriched at the same regions of PEPCK and G6Pase promoters relative to the intragenic regions in the fasted FL‐PGC‐1*α* ^−/−^ livers (Fig. 4E). No specific protein/DNA complexes were detected at the promoter and intragenic regions by immunoprecipitation with IgG in WT and FL‐PGC‐1*α* ^−/−^ samples (Fig. 4E). In contrast to the previous report by Boustead et al. (2003), neither FL‐PGC‐1*α*/NT‐PGC‐1*α* nor NT‐PGC‐1*α* ^254^ was enriched at the proximal HNF4*α* binding site (−76/−64) in the G6Pase promoter (data not shown). This might be due to weak or temporal formation of FL‐PGC‐1*α*‐ and/or NT‐PGC‐1*α*‐associated transcriptional complex on this region.

In agreement with fasting‐induced increase in PEPCK and G6Pase gene expression, FL‐PGC‐1*α* ^−/−^ mice exhibited normal fasting blood glucose levels after a 24 h‐fast (Fig. 4F, left panel). Basal and fasting blood insulin levels were also unaltered in FL‐PGC‐1*α* ^−/−^ mice (Fig. 4F, right panel). Collectively, these results suggest that fasting‐induced NT‐PGC‐1*α* ^254^ contributed to the elevated expression of PEPCK and G6Pase genes in FL‐PGC‐1*α* ^−/−^ mice upon glucose deprivation.

### NT‐PGC‐1*α* ^254^ is sufficient to activate fasting‐induced hepatic gluconeogenesis in FL‐PGC‐1*α* ^−/−^ mice

**Figure 5:**
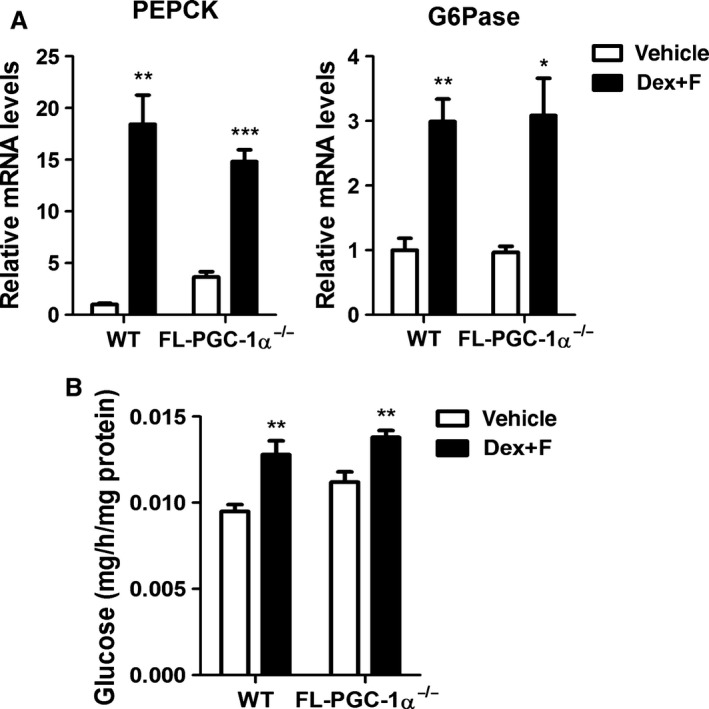
Gluconeogenic gene expression and glucose production are normal in FL‐PGC‐1*α* ^−/−^ primary hepatocytes. (A) Regulation of PEPCK and G6Pase gene expression in FL‐PGC‐1*α* ^−/−^ primary hepatocytes. Mouse primary hepatocytes were isolated from WT and FL‐PGC‐1*α* ^−/−^ mice and treated with vehicle or 1 μmol/L dexamethasone (Dex) and 10 μmol/L forskolin (F) for 2 h prior to qPCR analysis. (B) Normal glucose production by FL‐PGC‐1*α* ^−/−^ primary hepatocytes. WT and FL‐PGC‐1*α* ^−/−^ primary hepatocytes were treated with vehicle or 1 μmol/L dexamethasone (Dex) and 10 μmol/L forskolin (F) for 8 h and measured for glucose released into the medium. Data represent mean ± SEM. *P < 0.05; **P < 0.01; ***P < 0.001.

Lin et al. reported that PGC‐1*α* ^−/−^ mice lacking FL‐PGC‐1*α* and NT‐PGC‐1*α* have a significantly reduced ability to convert pyruvate to glucose during a pyruvate tolerance test (Lin et al. 2004). Forskolin/dexamethasone‐mediated induction of PEPCK and G6Pase expression is also greatly reduced in PGC‐1*α* ^−/−^ primary hepatocytes (Lin et al. 2004). To measure hepatic gluconeogenesis in FL‐PGC‐1*α* ^−/−^ mice, overnight fasted WT and FL‐PGC‐1*α* ^−/−^ mice were intraperitoneally injected with pyruvate that is the hepatic substrate for gluconeogenesis. The pyruvate bolus rapidly elicited a glycemic excursion in WT and FL‐PGC‐1*α* ^−/−^ mice, implying that FL‐PGC‐1*α* ^−/−^ mice produce glucose from pyruvate as efficiently as WT mice (Fig. 4G). To further determine whether NT‐PGC‐1*α* ^254^ is sufficient for activation of hepatic gluconeogenic program, we isolated primary hepatocytes from WT and FL‐PGC‐1*α* ^−/−^ mice and examined the expression of PEPCK and G6Pase genes in response to forskolin and dexamethasone. Basal levels of PEPCK and G6Pase mRNA were similar in WT and FL‐PGC‐1*α* ^−/−^ hepatocytes (Fig. 5A). Following treatments with forskolin and dexamethasone, WT and FL‐PGC‐1*α* ^−/−^ hepatocytes exhibited a strong induction of PEPCK and G6Pase gene expression (Fig. 5A). In addition, the increase in gluconeogenic gene expression was accompanied by increased glucose production in WT and FL‐PGC‐1*α* ^−/−^ hepatocytes (Fig. 5B). These data clearly demonstrate that NT‐PGC‐1*α* ^254^ is sufficient to promote gluconeogenic gene expression and hepatic glucose production in the absence of FL‐PGC‐1*α*.

### Fasting‐induced changes in hepatic gene expression are normal in FL‐PGC‐1*α* ^−/−^ mice

Fasting is a metabolic state that also increases fatty acid oxidation. An increase in fatty acid oxidation is required for generation of ATP that supports the enzymatic activity of the gluconeogenic pathway. In addition, increased oxidation of fatty acids augments the gluconeogenic flux by producing acetyl CoA necessary for the pyruvate carboxylase reaction and NADH necessary for the glyceraldehyde 3‐phosphate dehydrogenase reaction (Williamson et al. 1968; Ferre et al. 1979).

**Figure 6:**
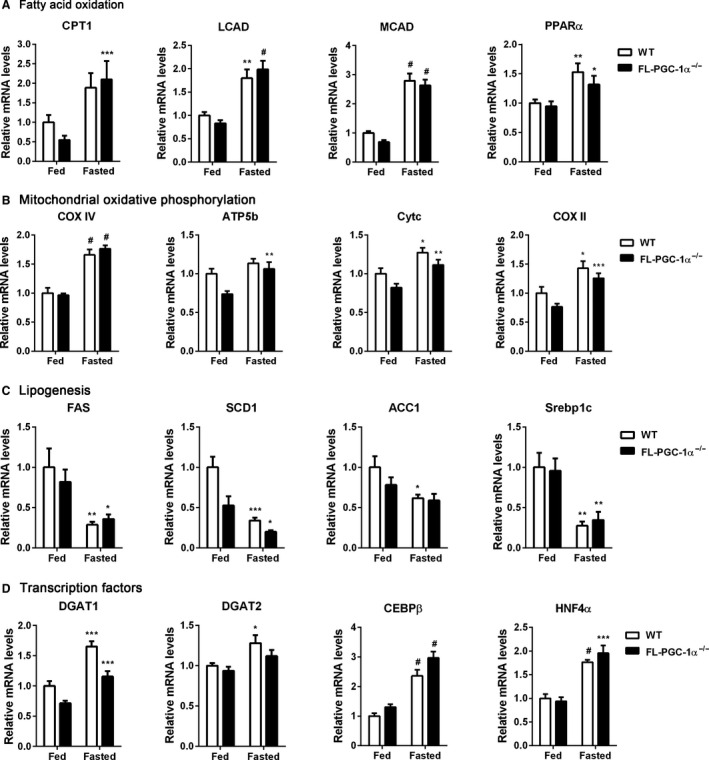
Hepatic gene expression profiles in FL‐PGC‐1*α* ^−/−^ mice. Quantitative real‐time PCR analysis of hepatic gene expression. Eleven‐week‐old WT and FL‐PGC‐1*α* ^−/−^ mice were fed ad libitum or fasted for 24 h before tissue collection (*n* = 8–10). Data represent mean ± SEM. *P < 0.05; **P < 0.01; ***P < 0.001; ^#^ *P* < 0.0001.

PGC‐1*α* is involved in fasting‐induced fatty acid oxidation in the liver (Yoon et al. 2001; Puigserver et al. 2003; Rhee et al. 2003). PGC‐1*α* stimulates fatty acid oxidation through its association with PPAR*α*. Since NT‐PGC‐1*α* (also NT‐PGC‐1*α* ^254^) can co‐activate PPAR*α* (Zhang et al. 2009; Chang et al. 2012), we evaluated the expression of fatty acid oxidation (FAO) genes in FL‐PGC‐1*α* ^−/−^ mice in response to fasting. Basal levels of FAO gene mRNA were similar in WT and FL‐PGC‐1*α* ^−/−^ mice (Fig. 6A). A 24 h fasting significantly elevated the expression of PPAR*α* in both genotypes. In addition, PPAR*α*‐target genes including CPT1*β*, LCAD, and MCAD were highly upregulated by fasting in WT and FL‐PGC‐1*α* ^−/−^ mice (Fig. 6A). The increase in FAO gene expression was concomitant with increased expression of mitochondrial genes involved in oxidative phosphorylation (COXIV, CYTC, and COXII) (Fig. 6B). In contrast, expression of lipogenic genes (FAS, SCD1, SREBP‐1c) was suppressed by fasting in WT and FL‐PGC‐1*α* ^−/−^ mice (Fig. 6C). In agreement with hepatic gene expression profiles, the FL‐PGC‐1*α* ^−/−^ livers did not show any histologic abnormalities under the fed and fasted conditions (data not shown).

## Discussion

Hepatic glucose production is a critical component of glucose homeostasis during prolonged fasting. The transcriptional co‐activator PGC‐1*α* plays a key role in fasting‐induced gluconeogenesis by linking hormonal and nutrient signals to the transcription program in the liver. We previously reported that fasting‐inducible NT‐PGC‐1*α* as well as full‐length PGC‐1*α* (FL‐PGC‐1*α*) are produced from the canonical promoter of PGC‐1*α* gene in the liver but no additional transcripts from the distal alternative promoter of PGC‐1*α* gene (Miura et al. 2008; Zhang et al. 2009). Here, we present the first insight of biological function of the truncated splice variant NT‐PGC‐1*α* in hepatic gluconeogenesis.

Adenovirus‐mediated expression of NT‐PGC‐1*α* in primary hepatocytes strongly induced the expression of PEPCK and G6Pase genes, leading to increased glucose production. Although the effect of NT‐PGC‐1*α* on PEPCK and G6Pase gene expression was smaller than that of PGC‐1*α*, glucose production driven by NT‐PGC‐1*α* and PGC‐1*α* was similar in NT‐PGC‐1*α*‐ and PGC‐1*α*‐expressing hepatocytes. This may be due to the fact that despite excessive PEPCK and G6Pase mRNA, glucose production is limited by the cellular capacity to generate energy (ATP). Our results clearly demonstrate that NT‐PGC‐1*α* is sufficient to activate the gluconeogenic program in primary hepatocytes. We think that NT‐PGC‐1*α* activates the promoters of PEPCK and G6Pase genes through interaction with HNF4*α* and GR, but not FOXO1. Indeed, our ChIP analyses demonstrated that hepatic NT‐PGC‐1*α* ^254^ is enriched at the PEPCK and G6Pase promoters containing binding sites for HNF4*α* and GR in FL‐PGC‐1*α* ^−/−^ mice. The in vitro studies further confirmed that NT‐PGC‐1*α* interacts with HNF4*α* and increases HNF4*α*‐mediated transcription of a reporter gene. HNF4*α* plays a crucial role in the regulation of PEPCK and G6Pase genes (Boustead et al. 2003; Rhee et al. 2003). HNF4*α* knockout abolishes PGC‐1*α*‐dependent induction of PEPCK and G6Pase gene expression in vivo (Rhee et al. 2003), and HNF4*α* but not FOXO1is required for PGC‐1*α*‐dependent activation of G6Pase gene expression in vitro (Boustead et al. 2003; Schilling et al. 2006). Although FOXO1 can be synergized by PGC‐1*α* (Puigserver et al. 2003), it can also independently induce gluconeogenic gene expression.

Adenovirus‐mediated PGC‐1*α* knockdown and liver‐specific PGC‐1*α* knockout, which diminish both FL‐PGC‐1*α* and NT‐PGC‐1*α*, greatly reduce fasting‐induced gluconeogenic gene expression and hepatic glucose production (Koo et al. 2004; Handschin et al. 2005). Likewise, whole‐body PGC‐1*α* knockout leads to impaired hepatic gluconeogenesis (Lin et al. 2004). In this mouse line, hepatic PEPCK and G6Pase gene expression is dysregulated due to abnormal upregulation of C/EBP*β*. In contrast, FL‐PGC‐1*α* ^−/−^ mice lacking FL‐PGC‐1*α* but expressing a functionally equivalent form of NT‐PGC‐1*α* (NT‐PGC‐1*α* ^254^) exhibited normal expression of C/EBP*β*, PEPCK and G6Pase in the fed and fasted states (Figs. 4D and 6D). Mechanistically, fasting‐induced NT‐PGC‐1*α* ^254^ was recruited to the PEPCK and G6Pase promoters with concomitant increase in fasting‐induced PEPCK and G6Pase gene expression, indicating that NT‐PGC‐1*α* ^254^ is sufficient to stimulate gluconeogenic gene expression in the absence of FL‐PGC‐1*α*. Moreover, loss of FL‐PGC‐1*α* function might be compensated by elevated levels of NT‐PGC‐1*α* ^254^ protein in FL‐PGC‐1*α* ^−/−^ mice. In agreement with normal expression of PEPCK and G6Pase genes, FL‐PGC‐1*α* ^−/−^ mice had normal fasting blood glucose levels and efficiently produced glucose from pyruvate during a pyruvate tolerance test. Previously, Burgess et al. reported conflicting results regarding the fasting response in FL‐PGC‐1*α* ^−/−^ mice. Six‐week‐old FL‐PGC‐1*α* ^−/−^ mice exhibited reduced hepatic glucose production in response to fasting (Burgess et al. 2006). However, this reduced ability to produce glucose was primarily due to diminished tricarboxylic acid cycle (TCA) flux, but not due to decreased gluconeogenic gene expression. We speculate that different findings between two groups are more likely related to age of mice subjected to a 24 h fast. Fasting requires high energy to support gluconeogenesis. Six‐week‐old FL‐PGC‐1*α* ^−/−^ mice may not generate sufficient energy to support gluconeogenesis since rapid growth and maturation are also occurring in this period. It has been shown that FL‐PGC‐1*α* ^−/−^ mice at 4–5 weeks of age exhibit temporary cold intolerance, whereas adult FL‐PGC‐1*α* ^−/−^ mice are able to defend against cold (Leone et al. 2005; Chang et al. 2012). Similarly, we observed no hepatic steatosis in adult FL‐PGC‐1*α* ^−/−^ mice after a 24 h fast and no alteration in hepatic gene expression profiles of fatty acid oxidation, mitochondrial oxidative phosphorylation, and lipogenic genes.

What are the physiological reasons for co‐expression of two different PGC‐1*α* isoforms in the liver? Although this study shows that NT‐PGC‐1*α* is recruited to the same target genes regulated by FL‐PGC‐1*α*, it does not address the relative contributions of NT‐PGC‐1*α* and FL‐PGC‐1*α* to target gene expression. We speculate that the timing of their action may be different during the course of fasting because protein stabilities of FL‐PGC‐1*α* and NT‐PGC‐1*α* are differentially regulated (Zhang et al. 2009). It is also possible that NT‐PGC‐1*α* and FL‐PGC‐1*α* may regulate a subset of isoform‐specific target genes in response to fasting. Genome‐wide occupancy analysis will be needed to elucidate whether there is differential recruitment to genomic sites between NT‐PGC‐1*α* and FL‐PGC‐1*α*. Our study also revealed that NT‐PGC‐1*α* and FL‐PGC‐1*α* activities are differentially regulated by insulin. Given that NT‐PGC‐1*α* gene expression is elevated in diabetic livers in ob/ob mice and that NT‐PGC‐1*α*‐mediated gluconeogenic gene expression is not suppressed by insulin, it is possible that NT‐PGC‐1*α* overexpression is implicated in the pathogenesis of hyperglycemia. However, in normal physiological conditions, insulin would indirectly repress NT‐PGC‐1*α* activity by inhibiting glucagon‐induced expression of PGC‐1*α* gene.

Collectively, this study demonstrates, for the first time, that hepatic NT‐PGC‐1*α* is a functional transcriptional co‐activator promoting gluconeogenic gene expression. Here, we propose that gluconeogenesis driven by NT‐PGC‐1*α*, along with PGC‐1*α*, contributes to the elevated blood glucose level in response to fasting.

## Conflict of Interest

None declared.
